# Circularly Polarized Luminescence Without External Magnetic Fields from Individual CsPbBr_3_ Perovskite Quantum Dots

**DOI:** 10.1021/acsnano.4c04392

**Published:** 2024-06-21

**Authors:** Virginia Oddi, Chenglian Zhu, Michael A. Becker, Yesim Sahin, Dmitry N. Dirin, Taehee Kim, Rainer F. Mahrt, Jacky Even, Gabriele Rainò, Maksym V. Kovalenko, Thilo Stöferle

**Affiliations:** † IBM Research Europe—Zurich, Säumerstrasse 4, 8803 Rüschlikon, Switzerland; ‡ Institute of Inorganic Chemistry, Department of Chemistry and Applied Biosciences, ETH Zurich, 8093 Zurich, Switzerland; § Laboratory for Thin Films and Photovoltaics, Empa, Swiss Federal Laboratories for Materials Science and Technology, 8600 Dübendorf, Switzerland; ∥ Université de Rennes, INSA Rennes, CNRS, Institut FOTON - UMR6082, 35000 Rennes, France

**Keywords:** perovskite, quantum dots, exciton fine-structure, circularly polarized luminescence, Stokes parameters, Rashba effect

## Abstract

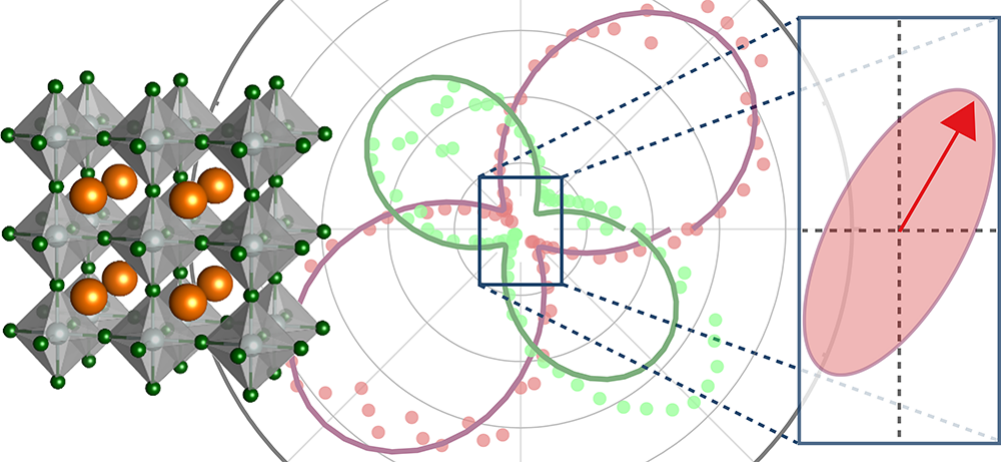

Lead halide perovskite quantum dots (QDs), the latest generation of the colloidal QD family, exhibit outstanding optical properties, which are now exploited as both classical and quantum light sources. Most of their rather exceptional properties are related to the peculiar exciton fine-structure of band-edge states, which can support unique bright triplet excitons. The degeneracy of the bright triplet excitons is lifted with energetic splitting in the order of millielectronvolts, which can be resolved by the photoluminescence (PL) measurements of single QDs at cryogenic temperatures. Each bright exciton fine-structure-state (FSS) exhibits a dominantly linear polarization, in line with several theoretical models based on the sole crystal field, exchange interaction, and shape anisotropy. Here, we show that in addition to a high degree of linear polarization, the individual exciton FSS can exhibit a non-negligible degree of circular polarization even without external magnetic fields by investigating the four Stokes parameters of the exciton fine-structure in individual CsPbBr_3_ QDs through Stokes polarimetric measurements. We observe a degree of circular polarization up to ∼38%, which could not be detected by using the conventional polarimetric technique. In addition, we found a consistent transition from left- to right-hand circular polarization within the fine-structure triplet manifold, which was observed in magnetic-field-dependent experiments. Our optical investigation provides deeper insights into the nature of the exciton fine structures and thereby drives the yet-incomplete understanding of the unique photophysical properties of this class of QDs for the benefit of future applications in chiral quantum optics.

Circular dichroism is the phenomenon of differential absorption of right- and left-hand circularly polarized light.^1^ The effect is observed in the absorption bands of chiral molecules, whose symmetry does not allow them to be superimposed on their mirror image.^2^ While circular dichroism probes the ground-state properties of materials, circularly polarized luminescence is defined as the right- or left-hand circularly polarized emission and provides information about the luminescent excited state.^3^ Circularly polarized emission and circular dichroism are found in inherently chiral systems like chiral molecules,^4,5^ chiral supramolecular assemblies,^6^ lanthanide ion complexes,^7^ transition metal complexes,^8^ chiral biomolecular systems,^9^ or chiral quantum dots (QDs)/rods.^10^ Moreover, chirality of an emitter can be induced by the attachment of chiral ligands^11,12^ or by a surrounding chiral matrix/solvent,^13^ leading to the preferred emission/absorption of left- or right-hand circularly polarized light. Differential light–matter interaction of left- and right-hand circularly polarized light can also be induced by static magnetic fields.^14^ While nonmagnetic methods are primarily used to investigate structural and stereochemical information, magnetically induced circular dichroism and especially circularly polarized luminescence can give important insight into the electronic structure of the emitters.^15^ In low-temperature magneto-optical measurements on single emitters, e.g., QDs, the type of polarization (linear or circular) elucidates optoelectronic fine-structure properties of excited states.^16−18^ Although chiroptical phenomena are extensively studied for fundamental research, there is also a growing interest in advanced photonic technologies that exploit the differential emission/absorption of right- and left-hand circularly polarized light,^5^ such as ellipsometer-based tomography^19^ or light-emitting diode (LED)^20^ and display technology.^21^ Moreover, circularly polarized luminescence of quantum emitters is central to the promising field of chiral quantum optics,^22^ with potential applications in quantum-information processing^23^ and quantum simulation.^24^

## Results and Discussion

### Material System

**Figure 1:**
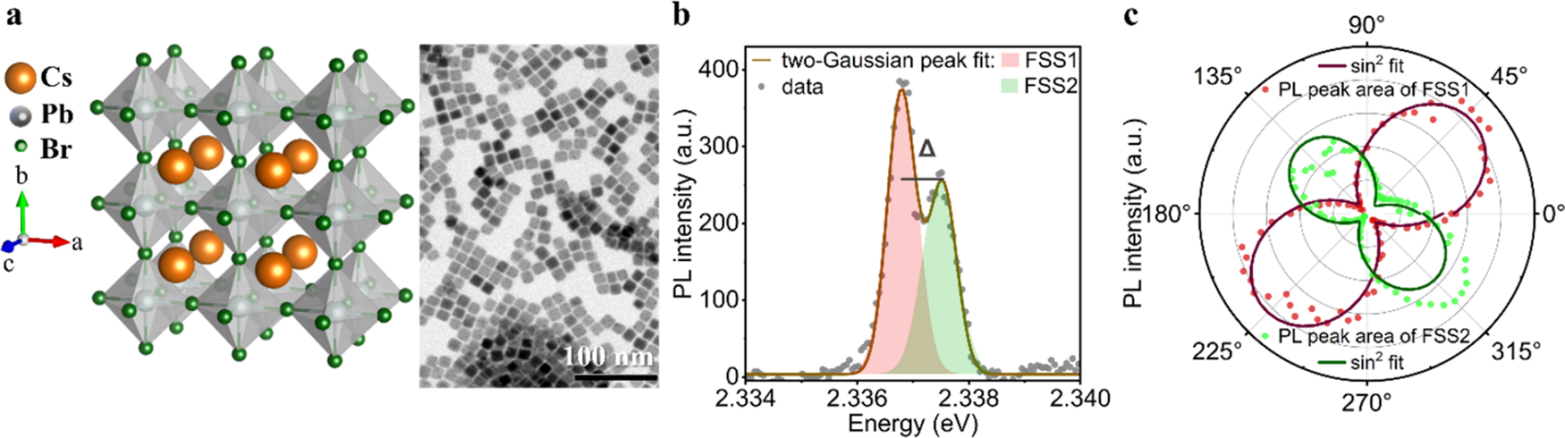
Crystal structure and emission properties of single cesium lead bromide QDs at 4 K. (a) Schematic crystal structure of CsPbBr_3_ QDs (left) and high-resolution transmission electron micrograph of CsPbBr_3_ QDs (right). (b) PL spectrum of a single CsPbBr_3_ QD at 4 K, recorded without polarization optics in the detection path. The brown solid line is the fit of two-Gaussian-peak function. This QD exhibits a doublet exciton with a splitting energy of Δ = 0.75 ± 0.08 meV. (c) Peak-area-intensity of each Gaussian fit as a function of linear polarizer angle for the QD displayed in panel (b). Solid lines represent sin^2^ fits to the data, revealing a DOLP of 82.9 ± 2.2 and 78.6 ± 4.8% for FSS1 and FSS2, respectively.

Cesium lead halide (CsPbX_3_) perovskite QDs are a rapidly emerging class of colloidal QDs due to their outstanding optoelectronic properties.^25−27^ The crystal structure of CsPbX_3_ perovskite is characterized by a three-dimensional network of corner-sharing PbX_6_ octahedra (X = Br, Cl, I) with the Cs^+^ filling the void, exhibiting an orthorhombic structure at low temperatures, as shown in the left panel of Figure 1a. With fluorescence quantum yields (QYs) approaching unity and a wide tunability of the emission wavelength,^28,29^ this type of QD can be utilized in various applications, such as quantum light sources,^30−32^ solar cells,^33^ lasers,^34^ displays,^35^ and even scintillators.^36^ Specifically at cryogenic temperatures, a nondegenerate bright triplet state with three orthogonal dipoles and high oscillator strength has been discovered.^18,26,37,38^ In addition, the CsPbBr_3_ QDs show a fast radiative lifetime (∼100 ps) with the exciton dephasing time on the order of several tens of picoseconds,^30,39−42^ which enables coherent light–matter interaction.^43^

A high-resolution transmission electron microscopy (HRTEM) image of one CsPbBr_3_ QD sample is displayed in the right panel of Figure 1a. These cuboidal QDs possess a side length of 14.0 ± 1.1 nm and a photoluminescence (PL) QY of 62% in solution at room temperature. In Figure 1b, we show an exemplary PL spectrum of a single CsPbBr_3_ QD at a cryogenic temperature (4 K), exhibiting doublet exciton fine-structure with an energy splitting of Δ = 0.75 ± 0.08 meV. As shown in many reports,^18,25,26,38,42,44,45^ fine-structure states (FSSs) of perovskite QDs possess a high degree of linear polarization, which is typically analyzed by tracking the transmitted PL intensity through a linear polarizer at varying angles. The result of such a measurement is depicted in Figure 1c (for the QD displayed in Figure 1b), where two sublevels exhibit a linear polarization profile along the crossed orientation. The solid lines represent the sin^2^ fits for the PL intensity trajectory of each FSS peak, from which we could extract the degree of linear polarization, *DOLP* = (*I*_max_ – *I*_min_)/(*I*_max_ + *I*_min_). For this QD, we obtained a DOLP of 82.9 ± 2.2 and 78.6 ± 4.8% for the low- and high-energy emission peaks (FSS1, FSS2), respectively. It should be noted that in many cases, the above formula is mistakenly used as a measure of the absolute degree of polarization. However, this is true only if the light does not possess any circularly polarized component. To distinguish between unpolarized and circularly polarized light, more sophisticated polarimetric techniques, e.g., Stokes polarimetric measurements, are required, from which the four Stokes parameters provide a complete description of different polarization states of light.

### Polarimetry Technique and Setup

**Figure 2:**
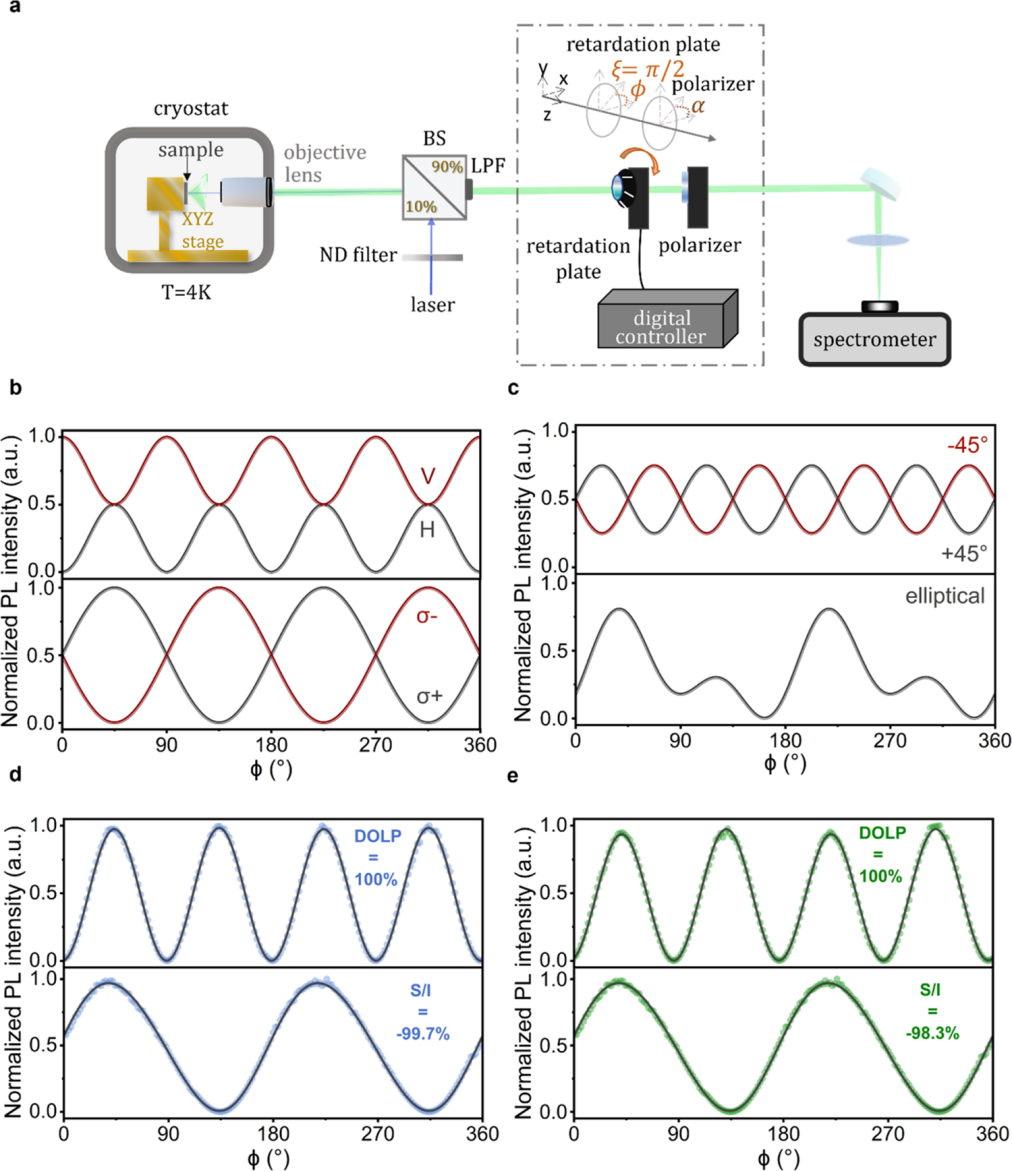
Experimental setup and examples of Stokes polarimetric measurements for various defined polarizations. (a) Schematic of the experimental setup. The inset depicts the basic measurement unit consisting of a λ/4*-*waveplate and a linear polarizer to perform Stokes polarimetric measurements. (b) Calculated intensity variation for fully linearly polarized light along the horizontal (H) and vertical (V) direction (upper panel) and right-hand (σ^–^) circularly polarized light (lower panel) as a function of the λ/4-waveplate angle, ϕ. (c) Calculated intensity variation for fully linearly polarized light along the +45/–45° direction (upper panel) and elliptically polarized light (lower panel) as a function of the λ/4-waveplate angle, ϕ. (d) Detected intensity variation as a function of λ/4-waveplate angle, ϕ. The defined linear (upper panel) and circular (lower panel) polarization are obtained from the laser light, generated with a combination of linear polarizer and λ/4-waveplate. Solid gray lines represent a fit of eq 1 to the data from which we retrieve a value of DOLP equal to 100% and a  of −99.7% for linear and circular polarization, respectively. (e) Detected intensity variation as a function of λ/4-waveplate angle, ϕ, for the linearly (upper panel) and circularly (lower panel) polarized laser light in panel (d) after going through our experimental setup. From the fit of eq 1 to the data, we obtain a DOLP of 100% and a value of  equal to −98.3%. Comparing panels (d, e), we prove that our experimental setup produces no or only negligible artifacts on the detected results.

In general, the polarization state of an electromagnetic field can be fully described by four measurable quantities known as Stokes parameters *I*, *M*, *C*, and *S*.^46,47^ To analyze the four Stokes parameters from the PL of our QDs, we used a combination of a quarter-waveplate (λ/4) and a linear polarizer in the detection path as shown in Figure 2a. As depicted in the inset of Figure 2a, ϕ is the angle between the vertical (*y*-axis) and the fast axis of the quarter-waveplate (λ/4) and α is the angle between the vertical (*y*-axis) and transmission axes of the polarizer. By measuring the transmitted light intensity while rotating a polarization optical element, one can determine the degree of linear and circular polarization, orientation, and handedness of the light field.^48^ To measure the Stokes parameters with the above-described setup, there are two equivalent techniques: measuring the transmitted intensity while rotating either the polarizer^49^ or the quarter-waveplate.^50^ We used the latter one because it is advantageous for not causing additional artifacts with the intrinsic polarization dependence of a grating-based spectrometer. Then, the detected intensity as a function of the quarter-waveplate angle, ϕ, is^48^
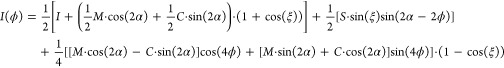
1Here, ξ is the retardation phase fixed as  (for the λ/4-waveplate), α is the angle of the polarizer, and *I* is the total intensity of the light beam. Correspondingly, the ratios  and  represent the degree of linear polarization in horizontal, vertical, and diagonal (+45, −45°) direction, respectively. Similarly,  represents the degree of circular polarization. In specific, the analyzed light is right-hand circularly polarized (RHCP) if  and left-hand circularly polarized (LHCP) if . Based on these Stokes parameters, the degree of linear polarization is defined as  and the total degree of polarization is defined as .

Based on eq 1, we could predict the intensity modulation for differently polarized light as a function of the λ/4-waveplate angle, ϕ. In Figure 2b, the calculated intensity modulations for fully linearly polarized light along the horizontal and vertical direction (upper panel) and fully left- (σ^+^) and right-hand (σ^–^) circularly polarized light (lower panel) are shown. Figure 2c shows the same for fully linearly polarized light along the +45/–45° direction (top panel) and elliptically polarized light (lower panel). For all cases, the degree of polarization was assumed to be 100% and exclusive (e.g., if DOLP = 100%, DOCP = 0%). The curves for purely circularly polarized light are π-periodic, whereas purely linearly polarized light exhibits a periodicity of π/2, which enables a clear distinction between the linear and circular polarization components. From the phase and intensity of the linear component, it is possible to extract the orientation and degree of linear polarization. Elliptically polarized light includes the components of both linear and circular polarization, and consequently, shows a combination of π/2- and π-periodicity. In particular, the elliptically polarized light shown exemplarily in Figure 2c is characterized by a degree of linear polarization of  and a degree of circular polarization of , with a total degree of polarization of DOP = 100%. Since , the circularly polarized component is left-handed.

A critical aspect to address before performing the polarimetric measurements is to test if the setup introduces any arbitrary modulation, e.g., unwanted birefringent phase shifts, occurring often when the light passes through optical coatings. In this regard, each optical element in the detection path should be tested in order to safely exclude experimental artifacts.^51,52^ For the setup calibration, we guided a laser beam at 532 nm (near the QD emission wavelength) with a well-defined polarization through the cryostat to mimic the optical path of the QD’s PL: through a microscope objective followed by all of the optical elements in the detection path, including beam splitter, long-pass filter, mirrors, and lenses. Consequently, the transmitted laser light was analyzed through a rotating λ/4-waveplate and a fixed polarizer as a function of the λ/4-waveplate angle, ϕ. For more details of the setup, see the Supporting Information. First, with a combination of a linear polarizer and λ/4-waveplate in the excitation path, we confirmed a light field with a well-defined linear and circular polarization prepared from our laser output (Figures 2d and S1a for the setup scheme). Next, we analyzed this well-defined light by guiding it through the optical components (PL detection path), as mentioned above (Figures 2e and S1b for the setup scheme). By fitting the intensity traces with eq 1, we could quantify the DOCP and DOLP from the Stokes parameters and compare these values before and after going through the detection path of our experimental setup. The prepared laser light originally exhibiting either near-unity DOLP (100%) or DOCP (99.7%) maintained the DOLP and showed only a very marginal change (1.4%) on DOCP after passing through the detection path, respectively. Thus, we validated that our setup introduces negligible modification to the light field passing through and that the polarization properties can be analyzed free from significant experimental artifacts (at least within the error range of ±1.4%).

### Single Quantum Dot Polarimetry

Hereafter, we investigated the polarization properties of the exciton FSSs of individual CsPbBr_3_ QDs at 4 K, utilizing a well-calibrated setup for Stokes polarimetric measurements. We found that these perovskite QDs, capped with nonchiral zwitterionic ligands, exhibit a substantial degree of circularly polarized emission that may reach up to ∼38%, which is technically not possible to detect via the standard polarimetric method. Our optical investigation provides deeper insights into different polarization components of the exciton fine-structure that have been poorly studied up to now, which may potentially enable applications in chiral quantum optics based on inexpensive, solution-processable, and wavelength-tunable all-inorganic perovskite QDs.

**Figure 3:**
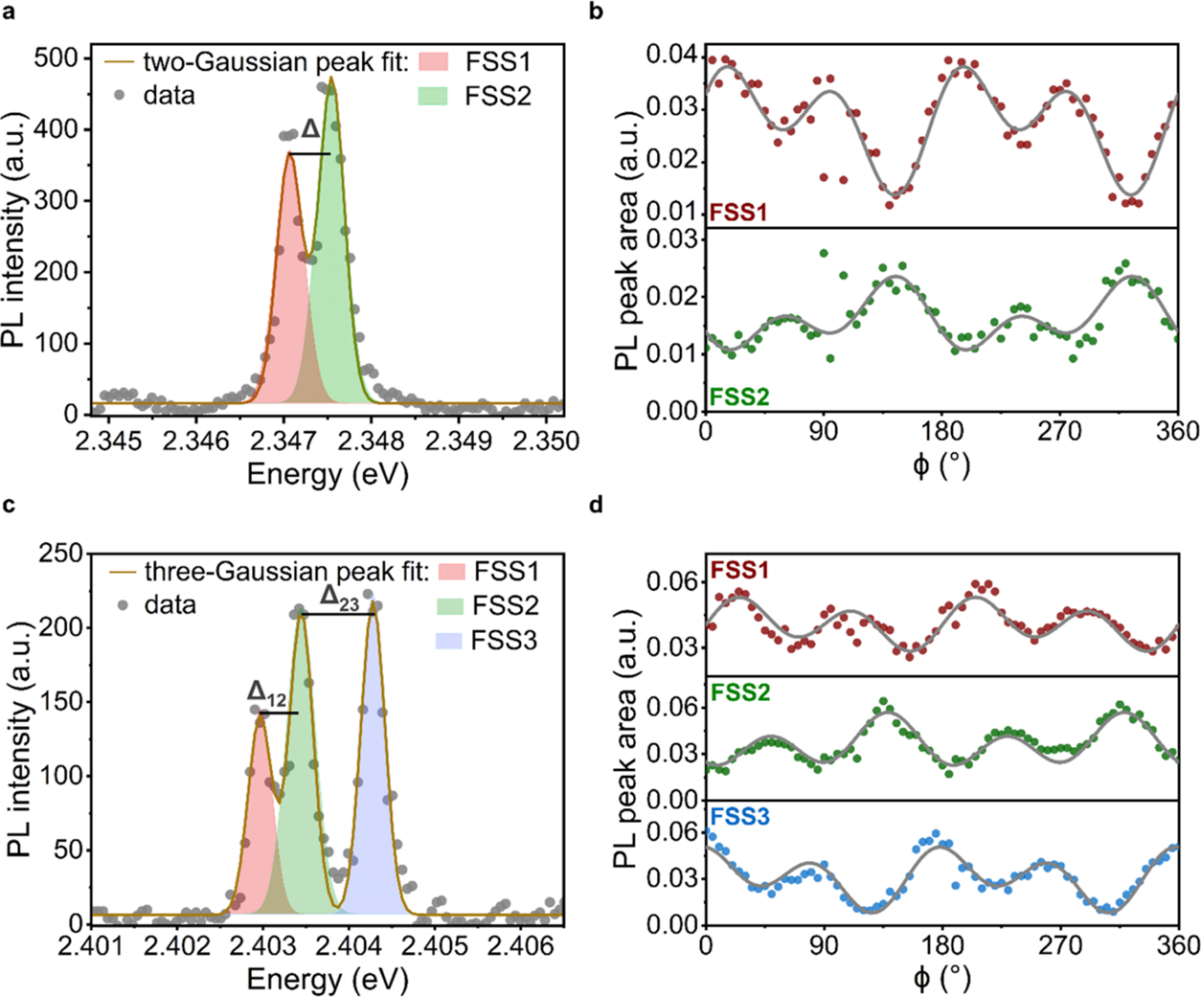
Stokes polarimetric measurements on exciton FSS in single CsPbBr_3_ QDs at 4 K. (a) Photoluminescence spectrum of a single CsPbBr_3_ QD exhibiting a doublet exciton FSS with a splitting Δ, recorded without polarization optics in the detection path. The brown solid line is a two-Gaussian peak fit to the data. (b) Peak-area-intensities of the doublet exciton fine-structure from Gaussian fits of the QD shown in panel (a), as a function of λ/4-waveplate angle, ϕ. Solid gray lines represent a fit of eq 1 to the data. (c) PL spectrum of a single CsPbBr_3_ QD exhibiting a triplet exciton fine-structure with splitting Δ_12_ and Δ_23_ between FSS1 and FSS2 and between FSS2 and FSS3, respectively, recorded without polarization optics in the detection path. The brown solid line is a three-Gaussian peak fit to the data. (d) Peak-area-intensities of the triplet exciton fine-structure from Gaussian fits of the QD shown in panel (c), as a function of λ/4-waveplate angle, ϕ, for FSS1 (upper panel), FSS2 (middle panel), and FSS3 (lower panel). Solid gray lines represent a fit of eq 1 to the data.

In Figure 3a,c, we show two examples of single CsPbBr_3_ QDs exhibiting clear FSSs, with two and three emission peaks, respectively, measured without a polarizer or λ/4-waveplate (see corresponding PL time series in Figure S2a,c). During the PL time-series measurements, on time scales of tens of seconds, we do not observe significant changes in the emission intensity due to fluorescence blinking,^25,53−55^ which ensures the solidity of Stokes polarimetric measurements. During the Stokes polarimetric measurements, to resolve all of the emission peaks and average out intensity fluctuations, we set an integration time of 5 and 10 s for the QD in Figure 3a,c, respectively. From low to high emission energies, we denote the exciton fine-structure states as FSS1 and FSS2 for doublet and FSS1, FSS2, and FSS3 for triplet. The QD exhibiting two emission peaks in Figure 3a showed a fine-structure splitting energy of Δ = 0.46 ± 0.03 meV. For the QD in Figure 3c, we observed the splitting energy between FSS1 and FSS2 of Δ_12_ = 0.50 ± 0.01 meV and the splitting energy between FSS2 and FSS3 of Δ_23_ = 0.84 ± 0.01 meV.

To analyze the polarization state of the individual emission peaks, we recorded the PL spectra as a function of ϕ (see Figure S2b,d) and fitted the evolution of peak intensity (integrated peak area) with respect to ϕ using multi-Gaussian functions with the full width at half-maximum (fwhm) of each peak as a shared free parameter for each individual QD. For example, for the two QDs displayed in Figure 3a,c, the fitted line widths (fwhm) were 0.21 ± 0.01 and 0.22 ± 0.01 meV, respectively (Figure 3b,d). From the traces, it is visible that all of the exciton FSSs of the two single QDs exhibit a noticeable degree of circular polarization (component of π-periodicity), especially in comparison to the purely linearly polarized laser light as displayed in Figure 2b. Similar results were observed for other individual QDs as well (Figures S3 and S4).

**Figure 4:**
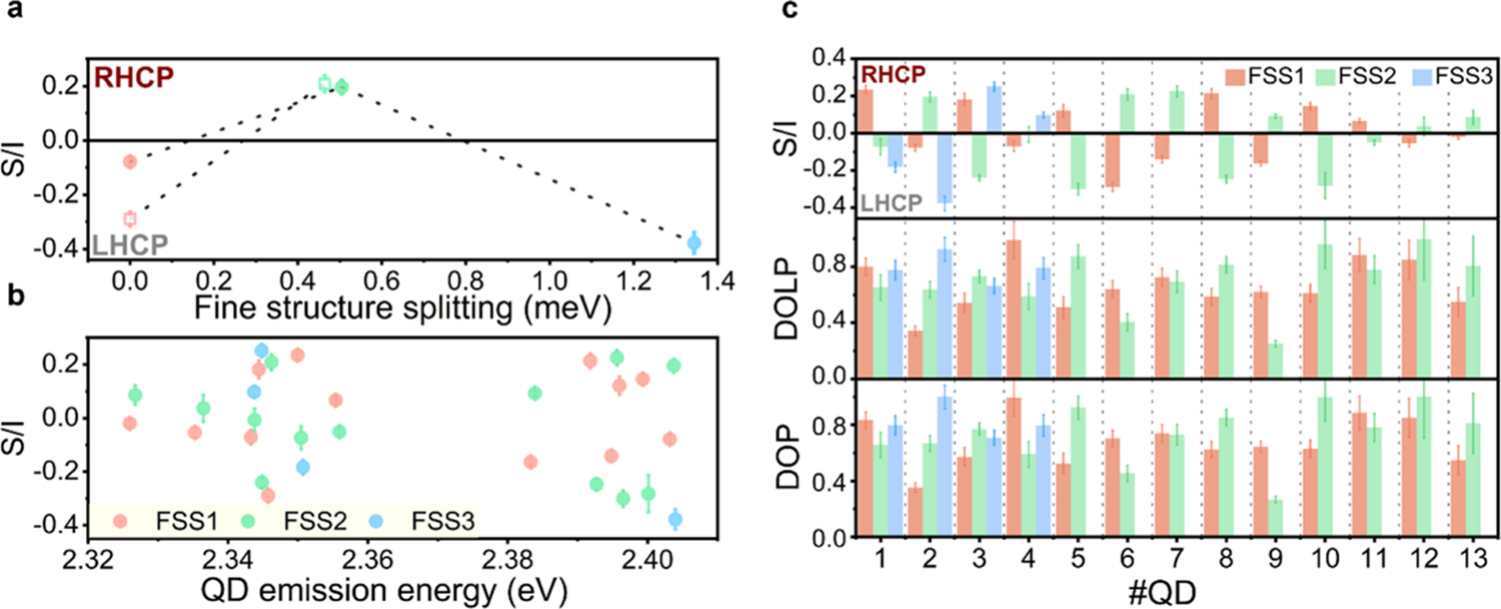
Measured Stokes parameters from each of the exciton fine-structure states in different single CsPbBr_3_ QDs. (a) Exemplary  values for two QDs exhibiting doublet (open squares) and triplet (filled spheres) exciton as a function of fine-structure splitting relative to FSS1, as displayed in Figure 3a–d, respectively. (b)  of all FSSs versus emission energy of individual QDs. (c) Extracted  values, degree of linear polarization (DOLP), and total degree of polarization (DOP) for all of the exciton fine-structure peaks in different QDs. The red bars correspond to FSS1, and the green bars correspond to FSS2. When the complete fine-structure triplet was resolved, the blue bar represents FSS3. QD number 2 and 6 are the QDs discussed in the main text. The error bars are obtained from the standard errors of the fit parameters when fitting eq 1 to the measured data.

To quantify the four Stokes parameters—*I*, *M*, *C*, and *S*—we fitted the peak intensity trace with eq 1, where we left the Stokes parameters as free parameters and constrained the polarizer angle, α, and the λ/4-waveplate phase, ξ, within a small range around the experimental values. For a reliable fit result, we carried out the global fit of emission peaks of each individual QD with a shared value of α and ξ among the FSSs. Figure 4a shows *S/I* values from all of the FSSs of the two QDs displayed in Figure 3, with respect to the energy difference of the fine structure peaks from FSS1. In principle, the detected light has a right-hand circularly polarized (RHCP) component if  and a left-hand circularly polarized (LHCP) component if . These two QDs show a degree of circular polarization  around 0.1–0.4. Over a wide range of QD emission energies (indicating QD of different sizes),  values remain rather constant, as shown in Figure 4b. Therefore, we conclude that all measured fine-structure emission peaks possess a non-negligible amount of circularly polarized component with an average value of . The handedness of the lowest FSS state is consistent with a random distribution, which is not surprising since the QDs are oriented randomly and no symmetry breaking external fields are present. Interestingly, however, there is the robust and nonrandom observation that  values change sign among FSSs, indicating that the handedness of circular component is flipped with respect to the adjacent energy state. The same observations apply to all of the studied QDs as shown in Figure 4b,4c (upper panel). The observation that within the same QD, some of the FSSs emit σ^+^(LHCP) light and others emit σ^–^(RHCP) light typically appears when external magnetic fields are present,^18,27^ which however was not the case in our experiments.

Furthermore, we calculated the degree of linear polarization (DOLP) and the degree of total polarization (DOP) and plotted them in the middle and lower panels of Figure 4c, respectively. FSS1, FSS2, and FSS3 are represented by red, green, and blue bars, respectively. Overall, we observed that the DOCP of single QD emission can reach up to ∼38%. As for the DOLP, it mostly ranged from 60% to at most 100%, consistent with many experimental studies based on standard polarization measurements, which reported a high degree of linear polarization, however, not always reaching 100%.^26,30,56^ Lower values of DOLP could be attributed to the unresolved peaks for doublets, low intensity-to-noise for the weak peaks within a triplet, and fluctuating emission intensity. The total degree of polarization (DOP) often reached near-unity values, with the average over all measured FSSs being 73 ± 19% (irrespective if doublet or triplet), nevertheless leaving a certain residual amount of unpolarized components. Furthermore, repetition of the Stokes measurement on the time scale of minutes gives very similar values (Figure S5 and Table S1), allowing us to exclude long-term polarization drifts.

### Circularly Polarized Emission Mechanism

In conclusion, our results prove that even in the absence of external magnetic fields, the single QD emission possesses a considerable portion of the circularly polarized component, in addition to the dominant linearly polarized component, that was already proposed by Isarov et al.^57^ in magneto-optical studies. We could exclude potential chromatic retardation effects introduced by our experimental setup, since we observed a clear sign change of  within an energy window of only a few millielectronvolts within the FSS manifold, after a complete calibration of our setup.

There are several possibilities for the observed circularly polarized PL in a QD. Circular dichroism and circularly polarized luminescence in QD could, for example, arise from the introduction of chiral ligand molecules that may impose their enantiomeric structure on the surface of the QD,^58−61^ which was also shown for perovskite QDs.^62^ Also, bulk and two-dimensional perovskite materials are known to exhibit circular dichroism if activated by chiral molecules.^63−66^ Furthermore, circular dichroism was observed in intrinsically chiral QDs, possibly due to the screw dislocations.^10^ We can safely exclude both the above-mentioned effects for the circularly polarized luminescence observed in this work since the QDs in our sample were stabilized with nonchiral zwitterionic ligands. Moreover, such effects do not explain the observed opposite handedness of the FSS emission lines. Neugebauer et al.^67^ showed that a linear dipole can possess a partially circularly polarized component in the nonpropagating near-field part of *k*-space. According to this study, if the evanescent longitudinal spin component^68^ was coupled from the near-field to a propagating wave by an optically denser medium, it should be detected as circularly polarized light in the far-field.^67^ However, the QDs in our measurement were embedded in a polystyrene layer, erasing an abrupt change of the refractive index at QD’s vicinity, which excludes this effect. Another possibility is that the coherent coupling of two resonant nonparallel dipoles would also lead to the emission of circularly polarized light. Since the FSSs are nondegenerate and (potentially) thermally populated (no coherent state), coherent coupling among the FSSs can also be ruled out. A similar effect with coherent coupling was also predicted to arise in QD molecules, where two resonant QDs were coupled.^69^ However, the simultaneous spectral diffusion of FSS peaks (see Figure S1a) proves that we were detecting single QDs and not two separate resonant QDs since the two separate QDs would exhibit independent spectral diffusion trajectories.

Meanwhile, a recent work^70^ stated that circular dichroism should also be observable in nonchiral metal halide perovskites due to a combination of an in-plane symmetry breaking, Rashba splitting, and the effect of the exciton momentum. As discussed earlier, the investigated QDs possess an edge length of ∼14 nm and therefore are in the so-called weak confinement regime. Hence, we do not expect the exciton to have a constant momentum that would induce circular dichroism or circularly polarized emission. On the other hand, the Rashba field itself could be responsible for the circularly polarized component of the fine-structure split states, as recently reported for layered perovskite compounds.^57,71,72^ The Rashba effect occurs as a result of an inversion-symmetry breaking in combination with strong spin–orbit coupling.^73^ In perovskite QDs, this effect was originally considered to be responsible for the large zero-field fine-structure splitting observed in cryogenic PL experiments.^26,37^ The [PbBr_6_]^4–^ structure is mainly responsible for the electronic band structure and therefore is also responsible for the strong spin–orbit coupling, whereas a displacement of the inorganic Cs^+^ ion can induce an inversion asymmetry.^74^ Charge carriers in perovskite materials should therefore experience a momentum-dependent effective magnetic field,^75^ which could result in mixing of bright and dark states^76^ or a reduction of the overall symmetry,^18^ even in the absence of an external magnetic field. This may explain why the detected PL of individual FSS was elliptically polarized and the observed change of polarization handedness within the FSS manifold with the lowest energy state emitting σ^–^-polarized light, similar to those results when the external magnetic field was applied.^18^ Moreover, if the displacement of Cs^+^ occurs randomly and relaxes faster than our measurement time scale (10 s integration time), this might justify the remaining, seemingly unpolarized emission fraction. An additional factor could be a reduction in the point symmetry at the level of individual QDs. *D*_2*h*_ (the point group related to *Pnma*) has *C*_2*v*_ as a potential point subgroup (4 symmetry operations instead of 8), which can induce circular dichroism. A quantitative description requires a more refined theoretical model, where the presented experimental results should serve as motivation and stimulus for further developments.

## Conclusions

In conclusion, we investigated the polarization properties of the bright exciton FSS at cryogenic temperatures by experimentally determining their Stokes parameters. In addition to the dominant linearly polarized component, the emitted light consistently exhibited a non-negligible fraction of circular polarization. Moreover, we observed both LHCP and RHCP light for different FSSs within a given QD. Our results provide an important puzzle piece to gain a complete understanding of the exciton fine-structure of perovskite QDs. Beyond the zero-field regime investigated here, future magnetic-field-dependent measurements of the Stokes parameters could provide additional insight into the evolution of bright triplet exciton states at higher fields. Furthermore, this elaborate Stokes polarimetric measurement technique can be applied to other types of (quantum) emitters^77,78^ or chiral nanostructures^79,80^ to close gaps and consolidate the understanding of their photophysical properties with potential applications in chiral quantum optics.

## Methods

### Chemicals

Cesium carbonate (Cs_2_CO_3_, 99.9%), 1-octadecene (ODE, 90%), 3-(*N*,*N*-dimethyloctadecylammonio) propanesulfonate (ASC18, >99.0%), and oleic acid (90%) were purchased from Sigma-Aldrich; lead(II) acetate trihydrate (>99%, for analysis) and bromine (Br_2_, >99%) were purchased from Acros Organics; trioctylphosphine (TOP) was purchased from Strem; toluene (99.85%, extra dry over molecular sieve, AcroSeal) was purchased from Thermo Scientific; and ethyl acetate (EtOAc, >99.7%, HPLC grade) was purchased from Fisher Scientific.

### Precursor Syntheses

#### Cesium Oleate

Cs_2_CO_3_ (1.628 g, 5 mmol) and oleic acid (5 mL, 16 mmol) were evacuated in a three-neck flask along with 20 mL of ODE at room temperature until the first gas evolution stops, heated to 120 °C under vacuum, and then further evacuated for 1 h at this temperature. This yielded a 0.4 M solution of Cs oleate in ODE. The solution turned solid when cooled to room temperature and was stored under nitrogen and heated before use.

#### Lead Oleate

Lead(II) acetate trihydrate (4.6066 g, 12 mmol) and oleic acid (7.6 mL, 24 mmol) were evacuated in a three-neck flask along with 16.4 mL of ODE at room temperature until the first gas evolution stops, heated to 120 °C under vacuum, and then further evacuated for 1 h. This yielded a 0.5 M solution of Pb oleate in ODE. The solution turned solid when cooled to room temperature and was stored under nitrogen and heated before use.

#### TOP-Br_2_

In a Schlenk flask, TOP (6 mL, 13 mmol) was dissolved in 18.7 mL of anhydrous toluene and Br_2_ (0.6 mL, 11.5 mmol) was added dropwise with vigorous stirring. The resulting solution was stirred for 1 h under a nitrogen atmosphere, forming a white-pale yellow viscous solution at the end. The solution (app. 0.46 M) was stored under nitrogen and heated before use.

### CsPbBr_3_ QD Synthesis

107.5 mg of ASC18, 2.0 mL of Cs oleate, 2.5 mL of Pb oleate, and 5 mL of ODE were added to a 100 mL three-neck round-bottom flask with a stir bar. The reaction mixture was evacuated and refilled with nitrogen 3 times and then heated to 180 °C under a nitrogen atmosphere, followed by the injection of 2.5 mL of TOP-Br_2_ with vigorous stirring. The resulting solution was rapidly cooled to room temperature using an ice–water bath and subjected to centrifugation at 12.1 krpm for 10 min. The initial precipitate obtained from this crude solution was size-fractioned in multiple cycles by adding various amounts of toluene for dispersion (table below). In each cycle, dispersion was centrifuged at 12.1 krpm for 10 min, followed by collecting the supernatant and redispersing the precipitate in toluene again. The supernatants from these cycles were denoted as pf_*n*_ (where *n* = 1, 2, 3, etc.), where for pf_1-3_ 1 mL of toluene was used and for pf_4-6_ 2 mL. The supernatant from the sixth cycle (pf_6_) was washed once with 2 equiv of EtOAc. The resulting precipitate was transferred directly into a glovebox and redispersed in 1 mL of anhydrous toluene. The final solution was filtered using a 0.45 μm syringe filter and used in the experiments.

### Sample Preparation

The single QD samples were prepared in a glovebox that is kept under a nitrogen atmosphere. The colloidal dispersion with a concentration of ∼1 mg/mL was diluted by a factor 100 in toluene (Acros Organics, 99.85% extra dry over molecular sieve). The solution was further diluted by another factor 100 in a 3-mass% solution of polystyrene (Aldrich, average *M*_*w*_ ∼ 280,000) in toluene, whereupon the solution was spin-coated at 3000 rpm for 60 s onto a crystalline Si wafer covered with a 3-μm-thick thermal oxide layer.

### Characterization of CsPbBr_3_ Solution

#### Absorption Spectra (UV–vis)

Optical characterizations were performed under ambient conditions. Ultraviolet–visible (UV–vis) absorption spectra of colloidal NCs were collected by using a Jasco V670 spectrometer in transmission mode.

#### Photoluminescence (PL)

A Fluorolog iHR 320 Horiba Jobin Yvon spectrofluorometer equipped with a PMT detector was used to acquire steady-state PL spectra. NC solutions were measured in the same dilutions and solvents as the absorption measurements.

#### Transmission Electron Microscopy (TEM)

The images were recorded using a JEOL JEM-1400+ microscope operated at 120 kV. Images were processed by using ImageJ.

### Single QD Optical Characterization

For single-QD spectroscopy, a home-built μ-PL setup was used. Samples were mounted on a *xyz* nanopositioning stage inside an evacuated liquid-helium closed-loop cryostat (MONTANA INSTRUMENTS) and cooled down to a targeted temperature of 4 K. Single QDs were excited by means of a fiber-coupled excitation laser, which was focused (Gaussian spot with 1/e^2^ diameter of 2.4 μm) on the sample by a dry microscope objective (NA = 0.8, 100×). Typical fluences used to excite single QDs were in the range of 2–6 nJ/cm^2^. The emitted light was collected by the same objective and passed through a 90:10 beam splitter and a long-pass filter at 500 nm. A monochromator coupled to a back-illuminated CCD (Princeton Instruments, 0.75 m) was used for recording the spectra. PL spectra were measured with a grating of 1800 lines/mm, with a blaze at 500 nm (0.2 meV spectral resolution). For the Stokes polarimetric measurement, we used a combination of a linear polarizer and retardation plate (λ/4-wave) in the detection path.

## Supporting Information Available

In the Supporting Information, we present details of the calibration procedure of the setup. We show the PL time series and the PL spectra of the QDs in the main text as a function of the retardation plate angle. Furthermore, we show PL spectra and their integrated emission as a function of the retardation plate angle for five more QDs (QD#1, QD#3, QD#5, QD#8, QD#10 in Figure 4). In order to check the absence of long-term polarization drift, we compare repeated measurements of two QDs (#1 and #10). The Supporting Information is available free of charge at https://pubs.acs.org/doi/10.1021/acsnano.4c04392.
