# Supplementary material for: Circularly Polarized Luminescence Without External Magnetic Fields from Individual CsPbBr3 Perovskite Quantum Dots
Source: ACS Nano. 2024 Jun 21;18(26):17218–27. doi: 10.1021/acsnano.4c04392 (PMC11223489; doi:10.1021/acsnano.4c04392)
Supplement: Supplementary file 1 — nn4c04392_si_001.pdf [file nn4c04392_si_001.pdf]

Supporting Information

Circularly Polarized Luminescence Without External Magnetic Fields from Individual $CsPbBr_3$ Perovskite Quantum Dots

Virginia Oddi[1,2,#], Chenglian Zhu[2,3,#], Michael A. Becker[1,$], Yesim Sahin[2,3], Dmitry N. Dirin[2,3], Taehee Kim[2,3], Rainer F. Mahrt[1], Jacky Even[4], Gabriele Rainò[2,3,],Maksym V. Kovalenko[2,3,*] Thilo Stöferle[1,*]*

[1] IBM Research Europe – Zurich, Säumerstrasse 4, 8803 Rüschlikon, Switzerland.

[2] Institute of Inorganic Chemistry, Department of Chemistry and Applied Biosciences, ETH Zurich, 8093 Zurich, Switzerland.

[3] Laboratory for Thin Films and Photovoltaics, Empa, Swiss Federal Laboratories for Materials Science and Technology, 8600 Dübendorf, Switzerland.

[4] Université de Rennes, INSA Rennes, CNRS, Institut FOTON - UMR6082, 35000 Rennes, France.

*** Corresponding Authors:** rainog@ethz.ch, mvkovalenko@ethz.ch, tof@zurich.ibm.com,

[#] These authors equally contributed to this work.

[$] Present address: Zeiss SMT, Oberkochen, Germany

Calibration of the setup

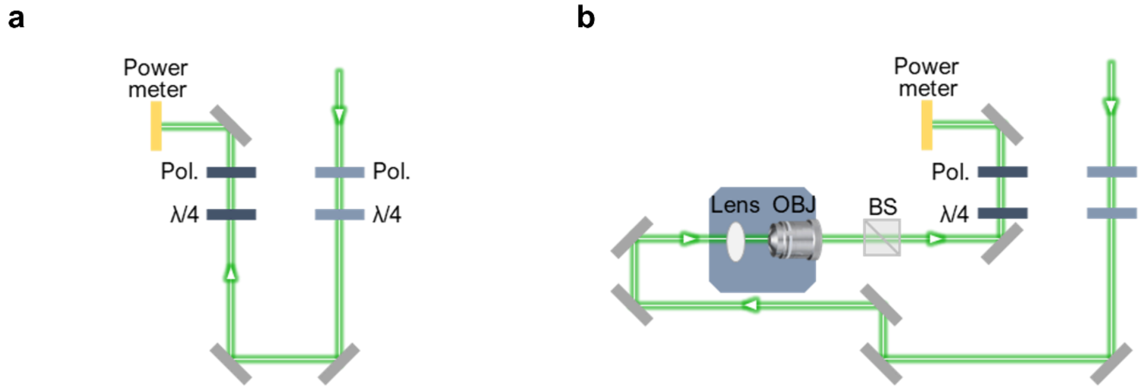

Figure S1: Calibration procedures of the polarimetric setup. a, A well-defined linear and circular polarization of the laser light was prepared by using either a linear polarizer or a combination of linear polarizer and λ/4-waveplate. The laser polarization state was analyzed through a rotating λ/4-waveplate and a fixed linear polarizer. Transmitted intensity as a function of the λ/4-waveplate angle, ϕ, was recorded by a power meter. **b,** We mimicked the PL optical path to assess the extent of modulation introduced by the optical components in the PL detection path. The polarized laser light set in **a** was sent through the cryostat, and then through all the optical components in the detection path of our setup (microscope objective lens, BS, LPF, mirrors, and lenses). Then, its polarization state was analyzed through a rotating λ/4-waveplate and a fixed polarizer. Again, the transmitted light intensity as a function of the λ/4-waveplate angle, ϕ, was recorded by a power meter.

2D plot of time-series and Stokes measurements

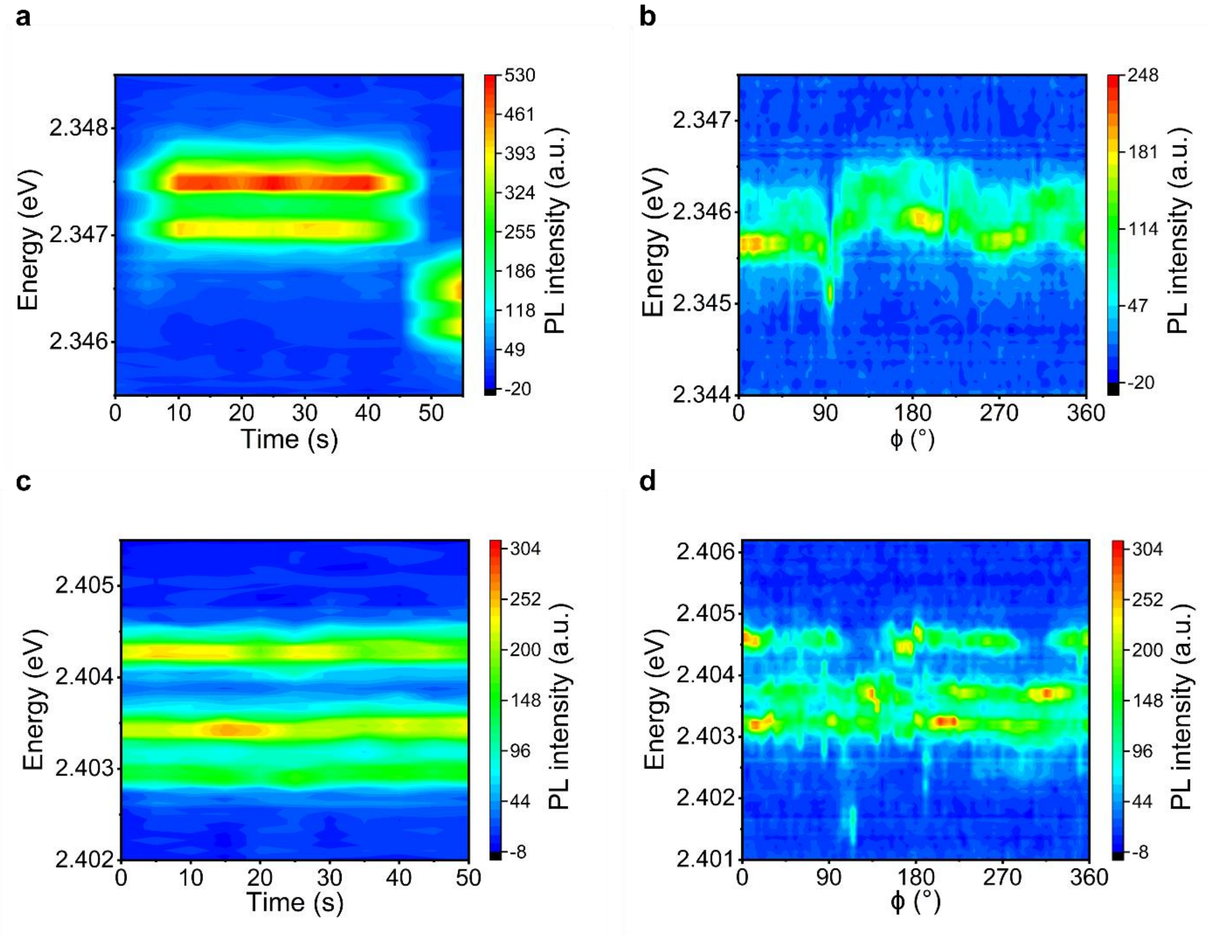

Figure S2: Time-series and spectral diffusion. a, PL time-series of the QD displayed in Figure 3a, acquired with an integration time of 5 s without polarization optics in the detection path.. **b,** PL spectra as a function of the λ/4-waveplate angle, ϕ, for the QD displayed in Figure 3a, acquired with an integration time of 5 s. **c,** PL time-series of the QD in Figure 3c, acquired with an integration time of 5 s without polarization optics in the detection path.. **d**, PL spectra as a function of the λ/4-waveplate angle, ϕ, of the QD in Figure 3c, acquired with an integration time of 10 s.

Fitting of Stokes parameters

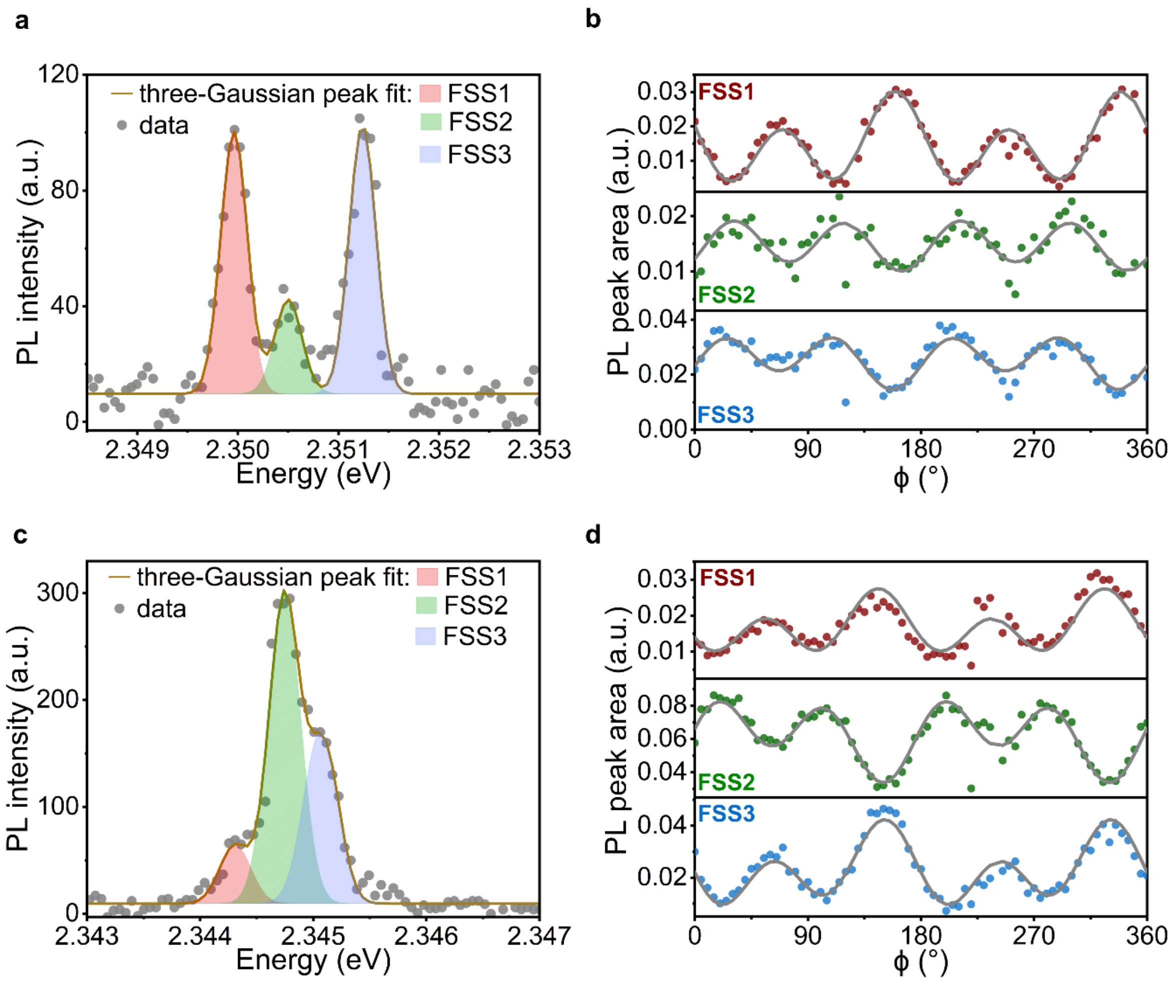

Figure S3: Stokes polarimetric measurements on QD#1 and QD#3 with triplet exciton. a, Spectrum of QD#1 in Figure 4. Triplet exciton is fitted with the sum of three Gaussian functions. **b**, Peak-area-intensity of the individual FSS of QD#1, extracted from the Gaussian peak fits, as a function of the λ/4-waveplate angle, ϕ. **c,** Spectrum of QD#3 in Figure 4. Triplet exciton is fitted with the sum of three Gaussian functions. **d**, Peak-area-intensity of the individual FSS in QD#3, extracted from the Gaussian peak fits, as a function of the λ/4-waveplate angle, ϕ. The spectra in a and c correspond to the data from b and d obtained at ϕ = 0°, respectively.

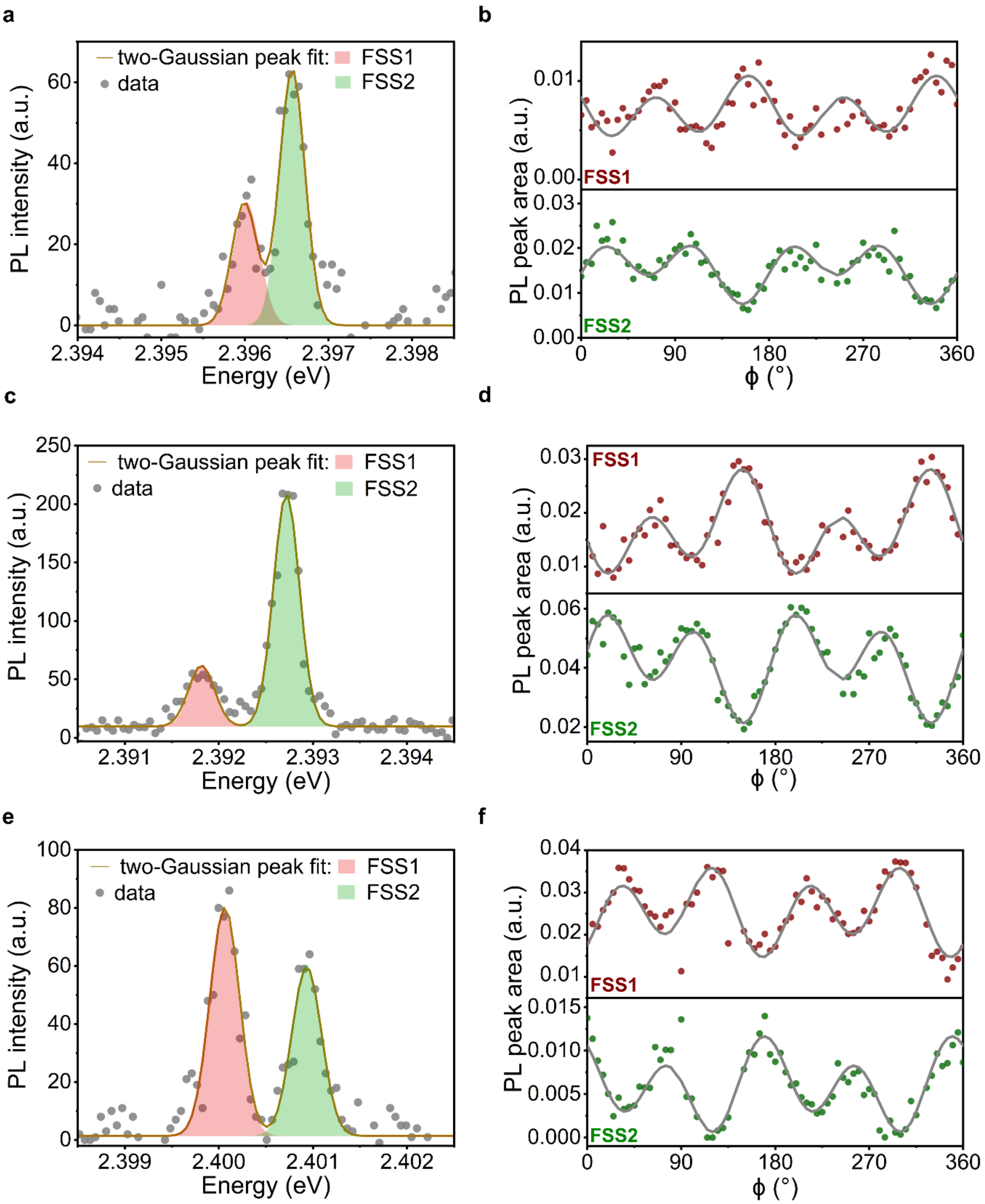

Figure S4: Stokes polarimetric measurements on QD#5, QD8 and QD#10 with doublet exciton. a, Spectrum of QD#5 in Figure 4. Doublet exciton is fitted with the sum of two Gaussian peaks. **b**, Peak-area-intensity of the individual fine-structure peaks in QD#5, extracted from the Gaussian peak fits as a function of the λ/4-waveplate angle, ϕ. **c** Spectrum of QD#8 in Figure 4. Doublet exciton is fitted with the sum of two Gaussian peaks. **d**, Peak-area-intensity of the individual fine-structure peaks in QD#8, extracted from the Gaussian peak fits as a

function of the λ/4-waveplate angle, ϕ. **e**, Spectrum of QD#10 in Figure 4. Doublet exciton is fitted with the sum of two Gaussian peaks. **f**, Peak-area-intensity of the individual fine-structure peaks in QD#10, extracted from the Gaussian peak fits as a function of the λ/4 angle, ϕ. The spectra in a,c and e correspond to the data from b, d and f obtained at ϕ = 0°, respectively.

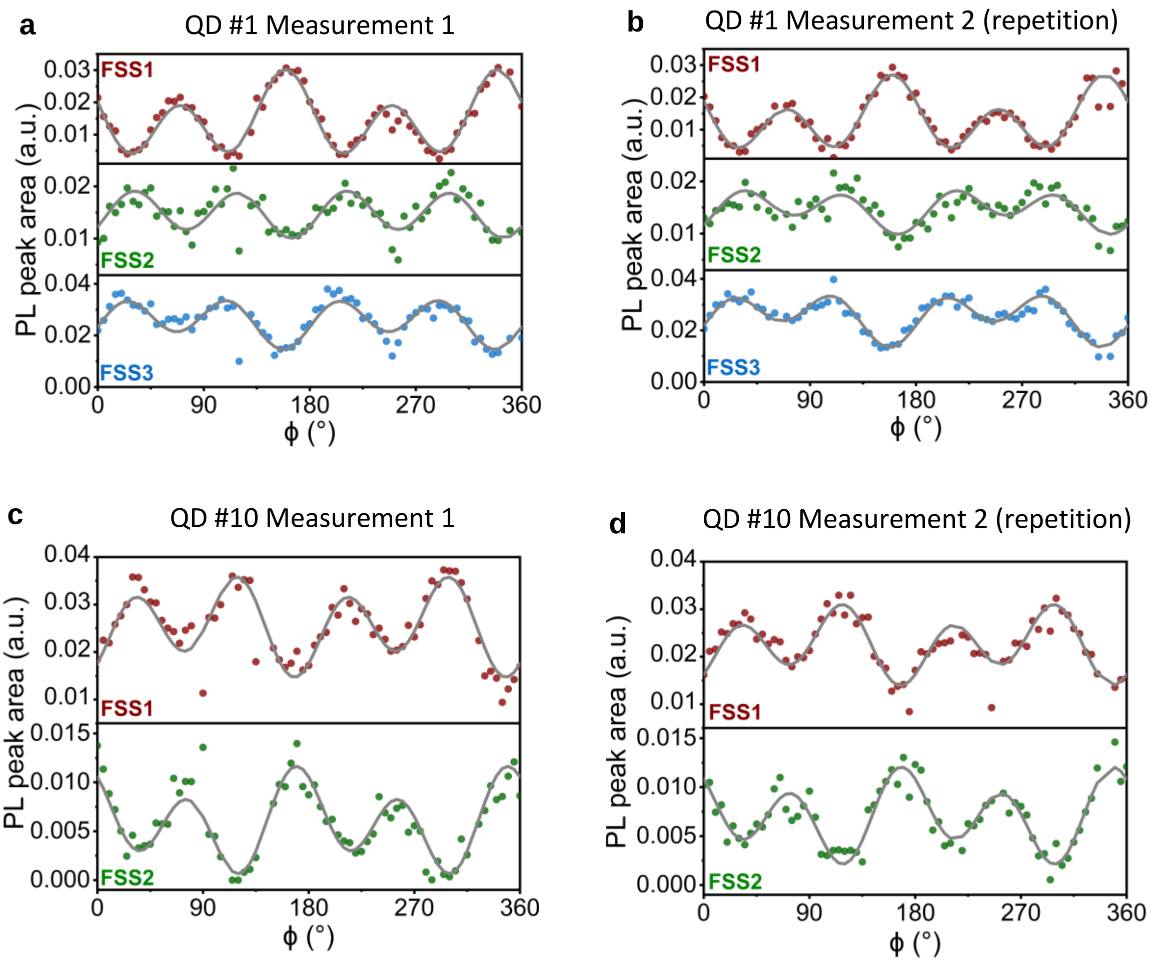

Figure S5: Absence of polarization "blinking". a,**b** Polarization traces of QD#1 with 5s integration time from two subsequent measurements, with tens of minutes between them (panel (a) is copied from Fig. S3a, for convenient side-by-side comparison). **c**,**d** Polarization traces of QD#10 with 10 s integration time from two subsequent measurements, with tens of minutes between them (panel (a) is copied from Fig. S4e, for convenient side-by-side comparison). Apart from singular outliers, no pronounced excursions from the fit are observed, excluding polarization fluctuations on the time scale of tens of seconds. The repetition measurements look very similar to the respective first measurements, indicating the absence of polarization fluctuations on the time scale of tens of minutes.

QD #1	Measurement 1			Measurement 2		
	FSS1	FSS2	FSS3	FSS1	FSS2	FSS3
S/I	0.24±0.02	-0.08±0.04	-0.19±0.03	0.25±0.02	-0.15±0.03	-0.26±0.02
M/I	-0.49±0.06	0.18±0.09	0.57±0.07	-0.48±0.05	0.14±0.06	0.54±0.05
C/I	-0.63±0.06	0.62±0.09	0.52±0.07	-0.60±0.04	0.46±0.06	0.45±0.05
DOLP	0.80±0.06	0.65±0.09	0.80±0.07	0.77±0.05	0.48±0.06	0.70±0.05
DOP	0.83±0.06	0.66±0.09	0.78±0.07	0.81±0.04	0.50±0.06	0.75±0.05

QD #10	Measurement 1		Measurement 2	
	FSS1	FSS2	FSS1	FSS2
S/I	0.15±0.02	-0.28±0.07	0.14±0.02	-0.26±0.05
M/I	-0.56±0.06	0.81±0.15	-0.50±0.04	0.87±0.12
C/I	-0.26±0.07	0.51±0.15	-0.14±0.05	0.30±0.11
DOLP	0.61±0.06	0.96±0.17	0.52±0.04	0.92±0.12
DOP	0.63±0.06	1.00±0.17	0.54±0.04	0.96±0.12

Table S1: Quantitative analysis of repetitive measurements of the same QDs. Here the results from the fits to the polarization traces in Fig. S5 are compared side-by-side. The deviations between them are consistent within the respectively given fit error margins, indicating the no substantial polarization changes are occurring in the time scale of tens of minutes.
